# Treatment Outcome and the Genetic Characteristics of Acute Promyelocytic Leukemia in Children in Poland From 2005 to 2018

**DOI:** 10.3389/fped.2020.00086

**Published:** 2020-03-20

**Authors:** Małgorzata Czogała, Katarzyna Pawińska-Wa̧sikowska, Teofila Ksia̧żek, Barbara Sikorska-Fic, Michał Matysiak, Anna Rodziewicz-Konarska, Alicja Chybicka, Jolanta Skalska-Sadowska, Jacek Wachowiak, Katarzyna Muszyńska-Rosłan, Maryna Krawczuk-Rybak, Dominik Grabowski, Jerzy Kowalczyk, Karolina Zielezińska, Tomasz Urasiński, Renata Tomaszewska, Tomasz Szczepański, Irena Karpińska-Derda, Mariola Woszczyk, Joanna Pohorecka, Grażyna Karolczyk, Wojciech Młynarski, Katarzyna Mycko, Wanda Badowska, Szymon Skoczeń, Walentyna Balwierz

**Affiliations:** ^1^Department of Pediatric Oncology and Hematology, Institute of Pediatrics, Jagiellonian University Medical College, Krakow, Poland; ^2^Department of Pediatric Oncology and Hematology, University Children Hospital, Krakow, Poland; ^3^Department of Medical Genetics, Institute of Pediatrics, Jagiellonian University Medical College, Krakow, Poland; ^4^Department of Pediatrics, Hematology and Oncology, Medical University of Warsaw, Warsaw, Poland; ^5^Department of Bone Marrow Transplantation, Pediatric Oncology and Hematology, Medical University of Wroclaw, Wroclaw, Poland; ^6^Department of Pediatric Oncology, Hematology and Transplantology, Poznan University of Medical Sciences, Poznan, Poland; ^7^Department of Pediatric Oncology and Hematology, Medical University of Bialystok, Bialystok, Poland; ^8^Department of Pediatric Hematology, Oncology and Transplantology, Medical University of Lublin, Lublin, Poland; ^9^Department of Pediatrics, Hematology and Oncology, Pomeranian Medical University, Szczecin, Poland; ^10^Department of Pediatrics Hematology and Oncology, Medical University of Silesia, Zabrze, Poland; ^11^Department of Pediatrics, Hematology and Oncology, City Hospital, Chorzów, Poland; ^12^Paediatric Department of Hematology and Oncology, Regional Polyclinic Hospital in Kielce, Kielce, Poland; ^13^Department of Pediatrics, Oncology, Hematology and Diabetology, Medical University of Lodz, Lodz, Poland; ^14^Department of Pediatrics and Hematology and Oncology, Province Children's Hospital, Olsztyn, Poland

**Keywords:** acute promyelocytic leukemia, children, variant translocations, treatment results, ATO, ATRA

## Abstract

**Background:** The aim of the study was to analyze the treatment outcome and genetic characteristics of acute promyelocytic leukemia (APL) in children in Poland from 2005 to 2018.

**Methods:** All 41 patients diagnosed with APL in Poland during the analysis period were eligible for the study. In period I (2005–2015), 33 patients were treated with chemotherapy and all-trans retinoic acid (ATRA), and in period II (2015–2018), 3 patients (high risk) received induction chemotherapy with ATRA and arsenic trioxide (ATO), and 5 patients (standard risk) received ATRA and ATO without chemotherapy.

**Results:** Probability of 5-years overall survival (OS), event-free survival (EFS), and relapse-free survival (RFS) was 0.819 ± 0.069, 0.831 ± 0.063, and 0.961 ± 0.037, respectively, in the whole cohort. Four (11%) early deaths were observed. One patient died of severe infection in the course of disease progression. Relapse occurred in one patient, who died finally because of disease progression. All events occurred in the patients from period I. Variant APL was identified in one patient (successfully treated with chemotherapy with ATRA) and complex translocation in one patient (the only patient with relapse). Additional chromosomal aberrations were found in 26% of patients and FLT3-ITD mutation was detected in 44% of patients; none of those changes influenced clinical outcome.

**Conclusion:** Treatment outcome in the analyzed group is similar to the results reported by other study groups. The main cause of death was coagulation disorders in the early stage of disease. Early, accurate diagnosis followed by specific treatment enables the reduction in the number of early deaths.

## Introduction

Acute promyelocytic leukemia (APL) is a specific subtype of acute myeloid leukemia (AML). In most cases, it is characterized by translocation (15;17) with the PML-RARA fusion gene (1–3), but about 1–2% of APL cases are due to rare variant translocations including ZBTB16/RARA, NMP/RARA, NUMA/RARA, STAT5B/RARA, PRKAR1a/RARA, BCOR/RARA, and FIP1L1/RARA (4–6). APL comprises about 5–10% of pediatric AML (1) and about 0.4% of all malignancies in children. Methods of the treatment used in children are based on clinical studies performed on greater adult population. Until the late 1980's, APL was the most lethal subtype of AML (2, 3). The result of the treatment improved significantly since the 1980's when specific treatment with all-trans retinoic acid (ATRA) was introduced (2, 3, 7–9). Since the 1990's, another specific drug—arsenic trioxide (ATO)—has been implemented. Efficacy and safety were first proven in adult patients (10–12) followed by the studies in children (1, 13–16). The combination of ATRA, ATO, and anthracycline-based chemotherapy ensures remission achievement in almost all patients (1, 13–16). Use of the specific treatment in APL allowed reduction of the chemotherapy especially cumulative anthracycline doses (13, 15–17). The main causes of the treatment failure are still early deaths, mostly in the course of intracranial hemorrhage (2, 3, 18). In the large analysis comprising 683 patients from different international studies, initial high WBC counts and obesity were found as likely predictors of thrombohemorrhagic early deaths in childhood APL (18).

Here, we present retrospective analysis of the treatment results of pediatric APL in Poland from 2005, when genetic analysis became widely available to confirm diagnosis of APL, to 2018. Children were treated according to two consecutive protocols, first (2005–2015) based on combination of chemotherapy and ATRA and second (2015–2018) based on ATRA and ATO with or without chemotherapy depending on number of leukocytes at diagnosis.

The aims of the study were to assess clinical outcome and determine the causes of treatment failures. We also performed analysis of additional genetic changes found in APL patients.

## Patients and Methods

From January 2005 to December 2018, 41 children (age 0–18) with newly diagnosed APL were treated in 16 centers of the Polish Pediatric Leukemia and Lymphoma Study Group. They comprised 6.5% of all 627 pediatric patients diagnosed with AML in that period. They were treated according to two consecutive protocols (AML-BFM 2004 Interim and AML-BFM 2012). Patient characteristics are summarized in Table 1. All of them were eligible for the study. The last patient was enrolled in May 2018 and the last follow-up was done in December 2018. Median observation time was 61.7 months (range, 7.0–145.5 months). The data were collected in Polish AML registry and analyzed retrospectively.

**Table 1:** Characteristics of the patients.

		**Total *n* = 41**	**Period I AML-BFM 2004 Interim (2005–2015) *n* = 33**	**Period II AML-BFM 2012 (2015–2018) *n* = 8**
Age, years		12.4	12.34	13.51
median (range)		(0.1–17.9)	(0.1–17.9)	(3.7–17.8)
Gender	Males	21 (51%)	18 (54.5%)	3 (37%)
	Females	20 (49%)	15 (45.5%)	5 (63%)
Observation time, months		61.7	61.7	18.4
median (range)		(7.0–145.5)	(12.7–145.5)	(7.0–30.0)

Informed consent to participation in the studies was obtained from guardians of all patients, in accordance with the Declaration of Helsinki. The study was approved by the Ethics Committee of Jagiellonian University Medical College.

All patients with characteristic bone marrow morphology and immunophenotype had diagnosis confirmed by the use of conventional cytogenetics, showing t(15;17) or variant translocations, and/or by fluorescence *in situ* hybridization tests for PML/RARa fusion or positive reverse transcription polymerase chain reaction (RT-PCR) assay.

The FLT3-ITD mutation analyses were routinely performed in all children. Patients were also screened for additional chromosomal aberrations.

**Figure 1:**
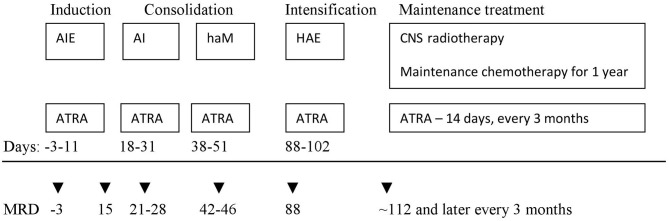
First period (AML-BFM 2004 Interim) treatment scheme. AIE—cytarabine 100 mg/m^2^/day [days 1–2] and 100 mg/m^2^ every 12 h [days 3–8], idarubicin 12 mg/m^2^/day [days 3, 5, and 7], and etoposide 150 mg/m^2^/day [days 6–8]. AI—cytarabine 500 mg/m^2^/day [days 1–4] and idarubicin 7.5 mg/m^2^/day [days 3 and 5]. haM—cytarabine 1 g/m^2^ every 12 h [days 1–3] and mitoxantrone 7 mg/m^2^/day [days 3–4]. HAE—cytarabine 3 g/m^2^/every 12 h [days 1–3], etoposide 125 mg/m^2^/day [days 2–5]. Maintenance chemotherapy—lasting 1 year: thioguanine 40 mg/m^2^/day orally, cytarabine 40 mg/m^2^/day intravenously for 4 consecutive days, every 4 weeks. ATRA—all trans-retinoid acid 25 mg/m^2^/day for 14 days. Intrathecal cytarabine in the age-depended dose (<1 year 20 mg; 1–2 years 26 mg; 2–3 years 34 mg; >3 years 40 mg) in each chemotherapy cycle and during maintenance treatment. CNS, central nervous system.

In the first period (January 2005–June 2015, period I) 33 patients were treated according to AML-BFM 2004 Interim Protocol; in the second period (July 2015–December 2018, period II) 8 children were treated according to AML-BFM 2012 Protocol. In period I, treatment consisted of four intensive chemotherapy cycles (AIE: cytarabine, idarubicine, etoposide, AI: cytarabine, idarubicine, haM: high-dose cytarabine, mitoxantron, HAE: high-dose cytarabine, etoposide; additional intrathecal cytarabine in every cycle) and maintenance therapy (6-thioguanine, cytarabine) for 1 year. All patients received ATRA concomitant with chemotherapy in 14-days cycles (Figure 1). Median observation time was 61.7 months (12.7–145 months).

**Figure 2:**
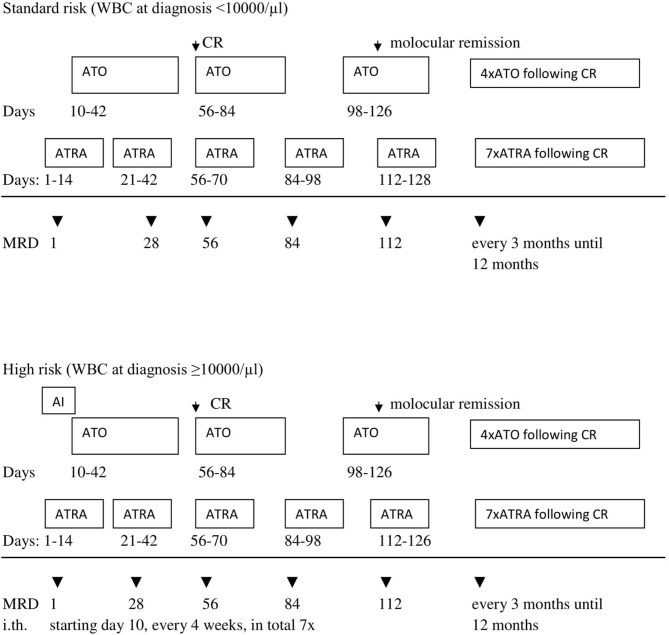
Second period (AML-BFM 2012) treatment scheme. AI—cytarabine 100 mg/m^2^/day [days 1–2] and 100 mg/m^2^ every 12 h [days 3–8], idarubicin 12 mg/m^2^/day [days 3, 5, and 7]. ATRA—all trans-retinoid acid 25 mg/m^2^/day oral in two divided doses for 14 days. ATO—arsentrioxide 0.15 mg/kg/day, starting at day 10 until morphologic CR, after 2 weeks brake: 4 cycles Monday–Friday 0.15 mg/kg/day i.v. for 4 weeks and 4 weeks break. MRD, minimal residual disease; i.th., intrathecal cytarabine in the age-depended dose (<1 year 20 mg; 1–2 years 26 mg; 2–3 years 34 mg; >3 years 40 mg). CR, complete remission.

In period II, according to the AML-BFM 2012 Protocol, patients with APL were classified into two groups according to initial number of white blood cells (WBC). There were five patients in the standard risk (SR) group with initial WBC <10,000/μl treated with the ATRA and ATO regimen. Three other patients were in the high-risk (HR) group (WBC more than 10.000/μl), and received one chemotherapy cycle (AI: cytarabine, idarubicine) with ATRA and ATO (Figure 2). Median observation time was 18.4 months (range, 7.0–30 months).

Molecular response was assessed centrally according to Europe Against Cancer program (19). It was monitored in period II, while the data from period I are very limited (complete data from 1 patient and two results in 5 patients−15 and 24 months from diagnosis). In period II and in 1 patient from period I, molecular minimal residual disease (MRD) was monitored on days 21, 56, 84, and 112 from the beginning of the treatment and then every 3 months for 12 months in the SR group and 18 months in the HR group.

In our database, we also collected data about differentiation syndrome (DS), severe adverse effect of ATO, and long-lasting consequences of the treatment in patients observed for at least 18 months. DS was defined as having at least three of the following symptoms: unexplained fever, weight gain, dyspnea, and pulmonary infiltrates.

The data that support the findings of the study are available on request from the corresponding author. The data are not publicly available due to privacy or ethical restrictions.

### Statistical Analysis

Descriptive statistical analysis was performed to assess patient baseline characteristics. We used Fisher's exact test or a chi-square test (categorical variables) and Mann–Whitney test (continuous variables) for analysis of clinical and laboratory features. Early mortality was defined as death within 42 days of the induction therapy. Overall survival (OS), event-free survival (EFS), and disease-free survival (DFS) were calculated using the Kaplan–Meier method. OS was defined as the time diagnosis to death from any cause; patients alive or lost to follow-up were censored at the date they were last known alive. EFS was defined as the time from diagnosis to disease progression, relapse, or death from any cause. Patients who were alive without disease progression or relapse were censored at the last time they were seen alive and event-free. DFS was defined as the time from complete remission (CR) to disease relapse, or death from any cause. Patients who were alive without disease relapse were censored at the time of last follow-up. For comparisons of Kaplan–Meier curves, we used the log-rank test. Because there were just eight patients in period II and observation time was much shorter than that in period I, we did not perform any statistical analysis to compare these two periods. All statistical analyses were performed using STATISTICA 12 software.

## Results

### Genetic Aberrations

In 27 patients (65.8%), complete cytogenetic analysis was available. Variant APL with t(11;17) (q23;q12) was identified in one patient and complex translocation involving chromosomes 4, 15, 16, and 17 in another patient (Table 2).

**Table 2:** Characteristics of the patients with additional genetical changes and variant APL.

	**Genetics**	**Age (years)**	**WBC (×10^**9**^/L)**	**PLT (×10^**9**^/L)**	**Complications**	**Outcome**	**Observation time (months)**
1	46,XY,t(11;17)(q23;q12)	<1	72.8	236	ND	Alive	14.7
2	46,XY,der(4)(4pter → 4q26::16q22 → 16qter),der(15)(15pter → 15q23::4q31.3 → 4qter),der(16)(16q22 → 16p11.2::17q25 → 17q21::16p11.2:: → 16pter)[20] PML/RARA(+), FLT3-ITD (–)	1–5	21.2	8	-	Death in course of progression after II relapse	37.7
3	45-47, XX,−6, add (6)(p25), t(5,17)(q22;q21),+mar, +mar (7)/46XX,PML/RARA(+),MLL(–),FLT3-ITD(–)	1–5	5.2	7	Subcutaneous and gingival bleeding	Alive	95.0
4	47,XX, +8, t(15;17)(q22;q21); PML/RARA(+) FLT3-ITD(–),	15–18	32.3	23	DIC, ATRA syndrome	Alive	35.0
5	46,XY,add(3),add(9),t(15;17),+mar; PML/RARA(+)	1–5	0.45	20	-	Alive	56.6
6	47XX,+8,t(15,17)(q22,q21)	10–15	2.0	187	ATRA syndrome	Alive	83.6
7	46,XY, t(15,17)(q22,q21)[9]/47XY+8; FLT3-ITD(–)	1–5	4.7	5	-	Alive	92.3
8	46XY, t(15;17)(q22.q21)[5]/46XY,t(15;17)(q22,q21), der(8)(pterq24…q11 qter), PML/RARA (+), FLT3-ITD(–)	13	20.0	24	-	Alive	61.7

*WBC, number of white blood cells at diagnosis; PLT, number of platelets at diagnosis; ND, no data; DIC, disseminated intravascular coagulation; ATRA, all trans-retinoic acid*.

Additional chromosomal aberrations were found in seven patients (26% of 27 patients with available karyotype results). We identified the trisomy of chromosome 8 in three patients, derivated chromosome 8 in one patient, complex karyotype in two patients (involving chromosomes 3 and 9 with marker chromosome in one patient and involving chromosome 6 with two marker chromosomes in one patient), and additional material from chromosome 16 in the above-described patient with complex translocation involving chromosomes 4, 15, 16, and 17 (Table 2).

Result of FLT3-ITD mutation analysis was available in 32 (78%) patients. This mutation was detected in 14 patients (44%).

### Treatment Response and the Treatment Failures

Thirty six (88%) patients achieved complete hematological remission. Data concerning molecular response were available in nine patients (one from period I and eight from period II). On day 21, molecular MRD was negative in two of seven patients with available result, and on day 56, it was negative in seven of eight patients with available results from that time point. All nine patients with available molecular monitoring had negative MRD results from day 84. No molecular relapse occurred. Four (9.7%) early deaths (5–10 days from diagnosis) caused by severe coagulation disorders were observed. Nine patients (21.9%) suffered from severe bleedings including those four children who died. One patient (2.4%) died of severe infection in the course of disease progression 1.9 months after diagnosis (no data concerning molecular MRD were available). Relapse occurred in one patient (2.4%), 17 months after first hematological remission. That was the patient with complex translocation. The patient did not respond to second-line chemotherapy (Idarubicine, Fludarabine) with ATRA, but achieved hematological remission after ATO treatment, followed by hematopoietic stem cell transplantation (HSCT). Fourteen months after HSCT, second relapse confirmed by molecular examination occurred, and the patient died 2 months later despite second-line therapy because of disease progression. No data concerning molecular response are available in that patient. Table 3 displays characteristics of the patients with the treatment failures. All events occurred in the patients from period I. There was no event in patients diagnosed after 2011 (21/41 children, 51%). Treatment results in two periods are presented in Table 4.

**Table 3:** Characteristic of the patients with the treatment failure.

	**Age (years)**	**WBC (×10^**9**^/L)**	**PLT (×10^**9**^/L)**	**Genetics**	**Events**	**EFS (months)**	**OS (months)**
1	10–15	40	26	46,XX,t(15;17)(q22;q21), PML/RARA(+), FLT3-ITD(+)	Early death (DIC, MOF)	0.17	0.17
2	1–5	151	0	PML/RARA (+), FLT3-ITD (+)	Early death (intracranial bleeding)	0.2	0.2
3	10–15	92	30	46,XY,t(15;17), FLT3-ITD non-available	Early death (intracranial bleeding)	0.3	0.3
4	1–5	21	8	46,XY,der(4)(4pter → 4q26::16q22 → 16qter),der(15)(15pter → 15q23::4q31.3 → 4qter),der (16)(16q22 → 16p11.2::17q25 → 17q21::16p11.2:: → 16pter) (20) PML/RARA(+), FLT3-ITD (–)	Relapse, death in progression after second relapse	17.3	37.7
5	10–15	24	6	46,XY,t(15;17), FLT3-ITD(–)	Death in progression	0	1.87
6	1–5	196	21	PML/RARA (+), FLT3-ITD analysis non-available	Early death (DIC, leukostasis)	0.33	0.33

*DIC, disseminated intravascular coagulation; MOF, multiorgan failure; WBC, number of white blood cells at diagnosis; PLT, number of platelets at diagnosis; EFS, event-free survival; OS, overall survival*.

**Table 4:** The treatment results in two consecutive periods.

	**Total *n* = 37**	**Period I AML-BFM 2004 Interim (2005–2015) *n* = 33**	**Period II AML-BFM 2012 (2015–2018) *n* = 8**
Complete remission (CR) achieved (%)	36 (87.8)	28 (84.8)	8 (100)
Early deaths (%)	4 (9.7)	4 (12.2)	0
Death in progression (%)	1 (2.4)	1 (3)	0
Relapse (%)	1 (2.4)	1 (3)	0
Continuous CR (%)	35 (85.3)	27 (81.8)	8 (100)
Severe bleedings (%)	9 (21.9)	8 (24.2)	1 (12.5)

### Analysis of Survival

**Figure 3:**
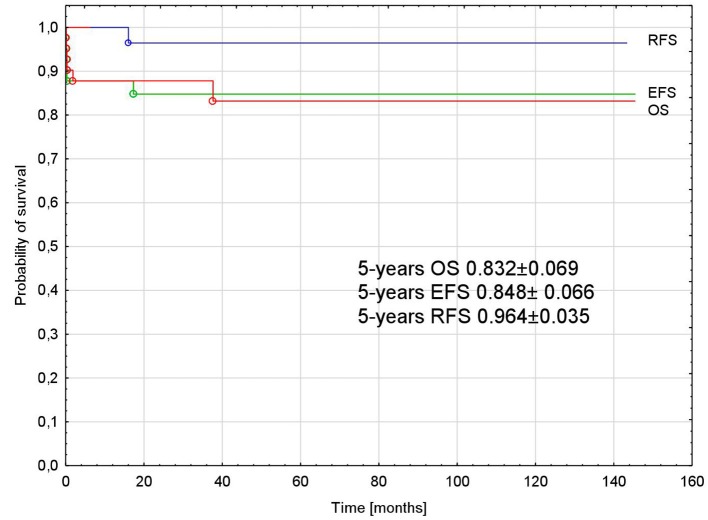
Probability of overall, event-free, and relapse-free survival in all analyzed patients with APL. APL, acute promyelocytic leukemia; OS, overall survival; EFS, event-free survival; RFS, relapse-free survival.

The probability of 5-years OS, EFS, and RFS was 0.832 ± 0.069, 0.848 ± 0.066, and 0.964 ± 0.035, respectively, for all analyzed patients (Figure 3).

White blood cell count at diagnosis was significantly higher in children who died early compared to the other patients (median [range]: 121 × 10^3^/μl [40–196 × 10^3^/μl] vs. 5.2 × 10^3^/μl [0.45–140 × 10^3^/μl], *p* = 0.003).

**Figure 4:**
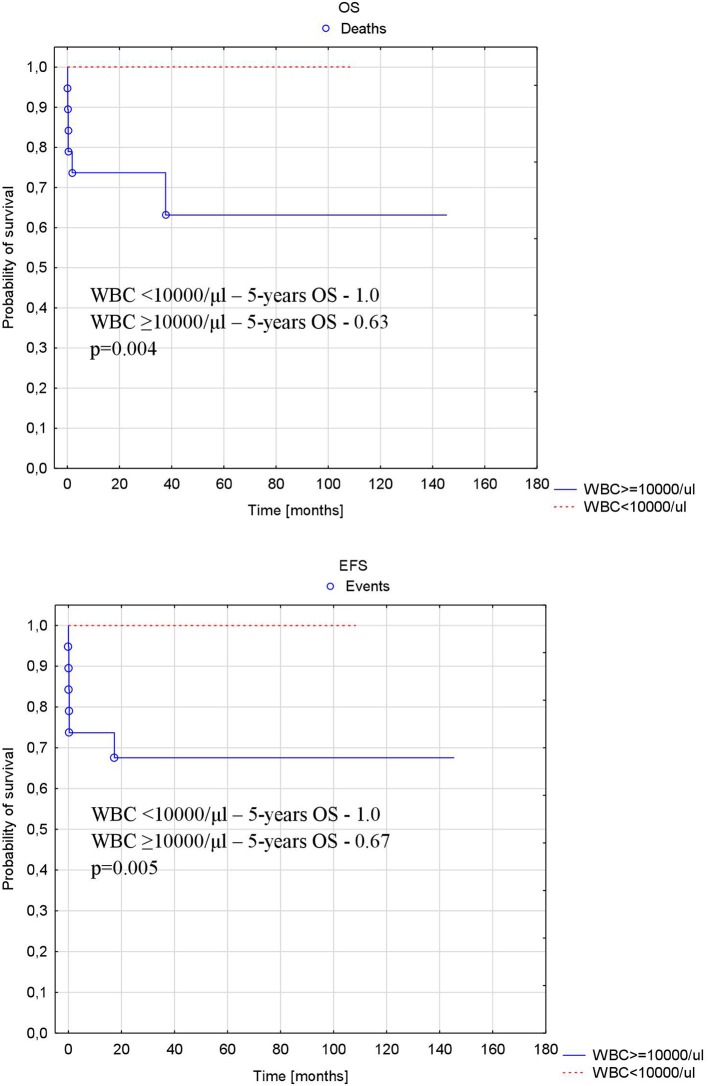
Probability of overall and event-free survival depending on number of WBC at diagnosis. OS, overall survival; EFS, event-free survival; WBC, white blood cell number at diagnosis.

Patients with more than 10,000/μl WBC at diagnosis had significantly lower probability of 5-years OS and EFS compared to patients with WBC <10,000/μl at diagnosis (1.0 vs. 0.63, *p* = 0.004 and 1.0 vs. 0.67, *p* = 0.005, respectively) (Figure 4).

The time from diagnosis to ATRA introduction did not differ between patients with treatment failure and other patients [median (range): 2 (1–4) days vs. 3 (0–56 days) *p* = 0.67].

There was no significant difference in OS, EFS, and DFS between patient FLT3-ITD positive and FLT3-ITD negative. Among 14 FLT3-ITD-positive patients, two early deaths occurred (14.3%), compared to 1 early death (5.5%), 1 progression (5.5%), and 1 relapse (5.5%) in 18 FLT3-ITD-negative patients. Number of WBC at diagnosis did not differ significantly in patients with and without FLT3-ITD mutation (median [range]: 18.2 × 10^9^/L [0.9–150.7 × 10^9^/L] vs. 6.5 × 10^9^/L [0.4–140 × 10^9^/L], respectively, *p* = 0.4).

Patients with additional chromosomal aberrations did not differ significantly from patients without those aberrations in terms of survival rates (OS, EFS, and RFS). The only event occurred in the patient with complex translocation and additional material from chromosome 16 who had two relapses and died of the disease progression 36.7 months after diagnosis.

The patient with variant translocation (11;17) was treated successfully with a combination of chemotherapy and ATRA and remains in remission with an observation time of 4 years.

### Adverse Events of ATRA and ATO

DS was observed in 29% of patients (9/31, no data from 10 patients), eight of whom were treated with steroids; in three patients, ATRA was held and then restarted.

There were no severe adverse events in patients treated with ATO. Transient rash occurred in one patient at the beginning of the therapy.

### Late Consequences of the Treatment

Data concerning long-lasting consequences of the treatment were available in 23 of 34 patients with an observation time of at least 18 months. One patient was diagnosed with aseptic bone necrosis and one was diagnosed with ischemic stroke of brain. We did not find any long-lasting toxicities in 21 patients (91%).

## Discussion

APL comprises about 5–10% of childhood acute myeloid leukemia (1). In the analyzed period, the percentage of the patients with APL among all children with AML in Poland was 6.5%. The treatment results achieved in the study group (OS, 0.832 ± 0.069; EFS, 0.848 ± 0.066; RFS, 0.964 ± 0.035) are comparable to the results described by other authors (1, 2, 7, 8, 13, 16, 17); however, the number of enrolled patients is relatively low. The data concerning molecular monitoring were limited in period I so comparison between two groups was not possible.

The bleeding complications remain the main cause of the treatment failures in APL. They were found in 4 (9.7%) patients in our cohort. It is worth noticing that there was no early death in patients with APL in Poland from 2011. It seems that early introduction of ATRA/ATO as well as oncology centers experience reduced the risk of early deaths. New treatment protocol introduced in 2015 with reduction of chemotherapy and use of ATO seems to be very effective. There were no events in that period; however, follow-up was rather short (median 18.4 months).

Variant translocation described as simple translocation involving chromosome 15 or 17 with any other chromosomes or complex translocations characterized by the involvement of additional chromosomes in addition to chromosomes 15 and 17 (4–6, 20–24) was found in two patients in the analyzed group. In one of them, t(11;17) (q23;q12) was identified, and in the second complex, translocation involving chromosomes 4, 15, 16, and 17 was found. The first patient was treated successfully with combination of chemotherapy and ATRA and remains in remission with an observation time of 4 years. The patient with complex translocation died of disease progression after second relapse.

Two different variant APL translocations involving chromosomes 11 and 17 were described before, t(11;17)(q23;q21) producing ZBTB16-RARA (formerly PLZF-RARA) fusion gene and t(11;17)(q13;q21) generating NUMA/RARA (20, 22). In cases of APL with t(11;17)(q23q21), there is evidence of resistance to ATRA and ATO both *in vivo* and *in vitro*, particularly in those patients who have the reverse rearrangement RARα-PLZF (20), while patients with t(11;17)(q13;q21) have better prognosis (22).

There are a number of studies concerning the alternate translocation in APL; however, little is still known about the complex variant translocations in APL (22, 23). Both 4, 15, 17, and 15, 16, 17 translocations have been described by other authors (4, 6, 22, 23), but the translocation found in our patient involving four chromosomes 4, 15, 16, and 17 has not been reported before.

In our cohort, additional chromosomal aberrations were found in 28% of patients with complete cytogenetic analysis, with the most common trisomy 8 (43%), similarly described by Cervera et al. (25). All but one patient from that group remain in CR. The only patient with treatment failure (death of disease progression after second relapse) was the child with complex translocation involving chromosomes 4, 15, 16, and 17 with additional material from chromosome 16. Analyzing all patients with additional chromosomal aberration in the study group, no differences in the treatment outcome were found compared to the patients with isolated t(15;17). It was reported by Cervera et al. that patients with and without additional chromosomal abnormalities had similar CR rates, and despite univariate analysis, they showed that additional chromosomal abnormalities were associated with a lower relapse-free survival in the LPA99 trial; such association was not present in the LPA96 trial. Neither additional chromosomal abnormalities overall nor any specific abnormality was identified as an independent risk factor for relapse in multivariate analysis (25).

In the current study, FLT3-ITD was present in 44% of patients. This is in accordance with the other studies where the frequency of FLT3-ITD mutation was 20–46% (26–30). No differences in WBC at diagnosis and the treatment outcome were found in patients with and without FLT3-ITD mutation but analyzed groups were rather small. The prognostic impact of FLT3-ITD mutation in APL remains controversial. Some authors describe negative influence on prognosis. Lucena-Araujo et al. screened for FLT3-ITD mutations in 171 APL patients (including nine children) and reported that FLT3-ITD mutations were associated with high WBC counts and may independently predict a shorter survival in patients with APL treated with ATRA and anthracycline-based chemotherapy (28). Association between FLT3-ITD mutation and high WBC was also confirmed by Barragan et al. in PATHEMA and HAVON groups, but the study did not demonstrate an independent prognostic value of FLT3-ITD mutation in patients with APL treated with ATRA and anthracycline-based regimens (29).

The COG AAML0631 study on childhood APL did not show an association of FLT3-ITD mutations with early death or bleeding/clotting events in induction; however, it was revealed that the relapse rate following ATO consolidation was significantly higher in FLT3-ITD mutant patients (30).

To conclude, the treatment outcome in children with APL in Poland is similar to results reported by other study groups. Reduction of early deaths in the last years was noticed. That could be the effect of introducing ATRA to a treatment protocol and the better experience of the study centers in the care for this challenging group of patients. A new treatment approach with the use of ATO and ATRA without chemotherapy in the SR group or with reduced chemotherapy in HR seems to be safe and effective. The limitation of the study is the relatively low number of enrolled patients. Further studies are needed to confirm the results with longer follow-up.

## Data Availability Statement

The datasets generated for this study are available on request to the corresponding author.

## Ethics Statement

The studies involving human participants were reviewed and approved by Bioethics Committee of Jagiellonian University Medical College. Written informed consent to participate in this study was provided by the participants' legal guardian/next of kin.

## Author Contributions

MC and WBal designed the study. MC, KP-W, BS-F, MM, AR-K, AC, JS-S, JW, KM-R, MK-R, DG, JK, KZ, TU, RT, TS, IK-D, MW, JP, GK, WM, KM, WBad, SS, and WBal were involved in participant recruitment. TK was involved in the laboratory work and interpretation of its results. MC, KP-W, BS-F, AR-K, JS-S, KM-R, DG, KZ, RT, IK-D, JP, and KM collected the clinical data. MC was involved in the statistical analysis and interpretation of its results, and wrote the first draft of the manuscript. WBal, KP-W, and SS edited the first draft of the manuscript. All authors reviewed the manuscript and approved the final version of the manuscript.

### Conflict of Interest

The authors declare that the research was conducted in the absence of any commercial or financial relationships that could be construed as a potential conflict of interest.

## References

[1] Kutny MA Gregory J Jr Feusner JH. Treatment of paediatric APL: how does the therapeutic approach differ from adults? Best Pract Res Clin Haematol. (2014) 27:69–78. 10.1016/j.beha.2014.04.007 24907019

[2] Stein EM Tallman MS Stein EM Tallman MS. Acute promyelocytic leukemia in children and adolescents. Acta Haematol. (2014) 132:307–12. 10.1159/000365117 25228556

[3] Zhang L Samad A Pombo-de-Oliveira MS Scelo G Smith MT Feusner J. Global characteristics of childhood acute promyelocytic leukemia. Blood Rev. (2015) 29:101–25. 10.1016/j.blre.2014.09.013 25445717 PMC4379131

[4] Redner RL. Variations on a theme: the alternate translocations in APL. Leukemia. (2002) 16:1927–32. 10.1038/sj.leu.2402720 12357344

[5] Brunel V Lafage-Pochitaloff M Alcalay M Pelicci PG Birg F. Variant and masked translocations in acute promyelocytic leukemia. Leuk Lymphoma. (1996) 22:221–8. 10.3109/10428199609051752 8819070

[6] Xu L Zhao WL Xiong SM. Molecular cytogenetic characterization and clinical relevance of additional, complex and/or variant chromosome abnormalities in acute promyelocytic leukemia. Leukemia. (2001) 15:1359–68. 10.1038/sj.leu.2402205 11516096

[7] Zwaan CM Kolb EA Reinhardt D Abrahamsson J Adachi S Aplenc R. Collaborative efforts driving progress in pediatric acute myeloid leukemia. J Clin Oncol. (2015) 33:2949–62. 10.1200/JCO.2015.62.8289 26304895 PMC4567700

[8] Fisher BT Singh S Huang YS Li Y Gregory J Walker D. Induction mortality, ATRA administration, and resource utilization in a nationally representative cohort of children with acute promyelocytic leukemia in the united states from 1999 to 2009. Pediatr Blood Cancer. (2014) 61:68–73. 10.1002/pbc.24585 23868668 PMC3927454

[9] Takahashi H Watanabe T Kinoshita A Yuza Y Moritake H Terui K. High event-free survival rate with minimum-dose-anthracycline treatment in childhood acute promyelocytic leukaemia: a nationwide prospective study by the Japanese paediatric leukaemia/lymphoma study group. Br J Haematol. (2016) 174:437–43. 10.1111/bjh.14068 27029412

[10] Lo-Coco F Avvisati G Vignetti M Thiede C Orlando SM Iacobelli S. Retinoic acid and arsenic trioxide for acute promyelocytic leukemia. N Engl J Med. (2013) 369:111–21. 10.1056/NEJMoa1300874 23841729

[11] Ghavamzadeh A Alimoghaddam K Rostami S Ghaffari SH Jahani M Iravani M. Phase II study of single-agent arsenic trioxide for the front-line therapy of acute promyelocytic leukemia. J Clin Oncol. (2011) 29:2753–7. 10.1200/JCO.2010.32.2107 21646615

[12] Breccia M Cicconi L Lo-Coco F. ATRA + ATO: has a new standard of care been established in low-risk acute promyelocytic leukaemia? Curr Opin Hematol. (2014) 21:95–101. 10.1097/MOH.0000000000000023 24434605

[13] Creutzig U Dworzak MN Bochennek K Faber J Flotho C Graf N. First experience of the AML-Berlin-Frankfurt-Münster group in pediatric patients with standard-risk acute promyelocytic leukemia treated with arsenic trioxide and all-trans retinoid acid. Pediatr Blood Cancer. (2017) 64:e26451. 10.1002/pbc.26461 28111878

[14] Cheng Y Zhang L Wu J Lu A Wang B Liu G. Long-term prognosis of childhood acute promyelocytic leukaemia with arsenic trioxide administration in induction and consolidation chemotherapy phases: a single-centre experience. Eur J Haematol. (2013) 91:483–9. 10.1111/ejh.12194 24033687

[15] Zhou J Zhang Y Li J. Single-agent arsenic trioxide in the treatment of children with newly diagnosed acute promyelocytic leukemia. Blood. (2010) 115:1697–702. 10.1182/blood-2009-07-230805 20029047

[16] Kutny MA Alonzo TA Gerbing RB Wang YC Raimondi SC Hirsch BA. Arsenic trioxide consolidation allows anthracycline dose reduction for pediatric patients with acute promyelocytic leukemia: report from the children's oncology group phase III historically controlled trial AAML0631. J Clin Oncol. (2017) 35:3021–9. 10.1200/JCO.2016.71.6183 28767288 PMC5590801

[17] Testi AM Pession A Diverio D Grimwade D Gibson B de Azevedo AC. Risk-adapted treatment of acute promyelocytic leukemia: results from the international consortium for childhood APL. Blood. (2018) 132:405–12. 10.1182/blood-2018-03-836528 29789356

[18] Abla O Ribeiro RC Testi AM Montesinos P Creutzig U Sung L. Predictors of thrombohemorrhagic early death in children and adolescents with t(15;17)-positive acute promyelocytic leukemia treated with ATRA and chemotherapy. Ann Hematol. (2017) 96:1449–56. 10.1007/s00277-017-3042-6 28597167

[19] Gabert J Beillard E van der Velden VH Bi W Grimwade D Pallisgaard N. Standardization and quality control studies of “real-time” quantitative reverse transcriptase polymerase chain reaction of fusion gene transcripts for residual disease detection in leukemia—a Europe against cancer program. Leukemia. (2003) 17:2318–57. 10.1038/sj.leu.2403135 14562125

[20] Piñán MA Balerdi A Iglesias A Acute Myeloid Leukemia with t(11;17)(q23;q21). Ann Hematol Oncol. (2015) 2:1050.

[21] Wang Y Ma J Liu X Liu R Xu L Wang L. A complex translocation (3;17;15) in acute promyelocytic leukemia confirmed by fluorescence *in situ* hybridization. Oncol Lett. (2016) 12:4717–19. 10.3892/ol.2016.5280 28101221 PMC5228300

[22] Zhang R Kim YM Wang X Li Y Pang H Lee JY. Coexistence of t(15;17) and t(15;16;17) detected by fluorescence *in situ* hybridization in a patient with acute promyelocytic leukemia: a case report and literature review. Oncol Lett. (2014) 8:1001–8. 10.3892/ol.2014.2304 25120648 PMC4114661

[23] Liu S Li Q Pang W Bo L Qin S Liu X. A new complex variant t(4;15;17) in acute promyelocytic leukemia: fluorescence *in situ* hybridization confirmation and literature review. Cancer Genet Cytogenet. (2001) 130:33–7. 10.1016/S0165-4608(01)00464-2 11672771

[24] He Y Wang P Liang K Chen X Du W Li J. A pediatric acute promyelocytic leukemia with a rare karyotype of ider(17)(q10)t(15;17) and favorable outcome: a case report. Medicine. (2015) 94:e1778. 10.1097/MD.0000000000001778 26469919 PMC4616798

[25] Cervera J Montesinos P Hernández-Rivas JM Calasanz MJ Aventín A Ferro MT. Additional chromosome abnormalities in patients with acute promyelocytic leukemia treated with all-trans retinoic acid and chemotherapy. Haematologica. (2010) 95:424–31. 10.3324/haematol.2009.013243 19903674 PMC2833072

[26] Gallagher RE Moser BK Racevskis J Poiré X Bloomfield CD Carroll AJ. Treatment-influenced associations of PML-RARα mutations, FLT3 mutations, and additional chromosome abnormalities in relapsed acute promyelocytic leukemia. Blood. (2012) 120:2098–108. 10.1182/blood-2012-01-407601 22734072 PMC3437597

[27] Kutny MA Moser BK Laumann K Feusner JH Gamis A Gregory J. FLT3 mutation status is a predictor of early death in pediatric acute promyelocytic leukemia: a report from the children's oncology group. Pediatr Blood Cancer. (2012) 59:662–7. 10.1002/pbc.24122 22378655 PMC3368997

[28] Lucena-Araujo AR Kim HT Jacomo RH Melo RA Bittencourt R Pasquini R. Internal tandem duplication of the FLT3 gene confers poor overall survival in patients with acute promyelocytic leukemia treated with all-trans retinoic acid and anthracycline-based chemotherapy: an international consortium on acute promyelocytic leukemia study. Ann Hematol. (2014) 93:2001–10. 10.1007/s00277-014-2142-9 24981688

[29] Barragán E Montesinos P Camos M González M Calasanz MJ Román-Gómez J. Prognostic value of FLT3 mutations in patients with acute promyelocytic leukemia treated with all-trans retinoic acid and anthracycline monochemotherapy. Haematologica. (2011) 96:1470–7. 10.3324/haematol.2011.044933 21685470 PMC3186308

[30] Kutny MA Alonzo TA Gerbing R Wang Y-C Fu C Aiu X FLT3 mutations in pediatric acute promyelocytic leukemia; a report from the children's oncology group AAML0631 trial. Blood. (2016) 128:2884 10.1182/blood.V128.22.2884.2884

